# Biomolecules from Different Angles

**DOI:** 10.3390/biom11010014

**Published:** 2020-12-26

**Authors:** Vladimir N. Uversky

**Affiliations:** 1 Department of Molecular Medicine, Morsani College of Medicine, University of South Florida, Tampa, FL 33612, USA; vuversky@usf.edu; Tel.: +1-831-974-5816; 2 Laboratory of New Methods in Biology, Institute for Biological Instrumentation, Russian Academy of Sciences, Federal Research Center “Pushchino Scientific Center for Biological Research of the Russian Academy of Sciences”, 142290 Pushchino, Russia

Special Issue “2019 Feature Papers by *Biomolecules*’ Editorial Board Members” represents a set of papers based on the results of the research in the laboratories of the Editorial Board Members (EBMs) of *Biomolecules* focused (a big surprise!) on different aspects of biomolecules. Therefore, this Special Issue is a snapshot of a collective view of *Biomolecules* on biomolecules. The covered topics range from the description of secondary metabolites produced by fungi [1] to the overview of the phytochemical composition and pharmacological and food applications of *Prosopis* plants [2], to comparative evaluation of the antiproliferative activity of plant-derived silver nanoparticles synthesized using callus and anthocyanin extracts of purple basil (BC-AgNPs and AE-AgNPs, respectively) [3], to systematic description of the utilization of natural products as a source of antitumor drugs [4], to the analysis of the effects of caffeine and other methylxanthines on Aβ-homeostasis by shifting the α- and β-secretase-based processing of amyloid precursor protein (APP) from the Aβ-producing amyloidogenic to the non-amyloidogenic pathway [5], to the topoisomerase I and radical trapping antioxidant activities of a series of N-alkyl-acridones and N,N′-dialkyl-9,9′-biacridylidenes [6], to the analysis of the effects of phytoalexins in soybeans via synergistic inhibition on α-glucosidase [7], to functional and structural characterization of several important proteins (such as the analysis of the general and genomic DNA-binding specificity of a transcription factor TTHB023 from *Thermus thermophilus* HB8 [8], investigation of the voltage sensing in protein translocation via the bacterial channel SecYEG [9], and structural characterization of the terminase (packaging protein) pUL15, which is the most conserved among all the Herpes Simplex Virus 1 (HSV1) gene products [10]), to the description of utilization of kinetic transition in amyloid assembly for screening fluorophores for preferential responses to oligomer over fibril formation [11], to the description of the utilization of amyloid fibril biomaterials, designer amyloid cell-penetrating peptides comprised of β-sheet cores derived from naturally occurring protein sequences and designed positively charged and aromatic residues exposed at key residue positions as gene transfer vehicles capable of self-assembling and promoting the DNA condensation and cell internalization [12], to the introduction of the use of phage display system for generation of lamprey monoclonal antibodies, lampribodies, which are the variable lymphocyte receptors (VLRs) consisting of leucine rich repeats (LRRs), whose diversity is generated by stepwise genomic rearrangements of LRR cassettes dispersed throughout the VLRB locus [13], to the description of changes in the N-glycomic profile associated with post-traumatic stress disorder (PTSD) via comparative analysis of the N-glycomic profiles in 543 male Caucasian individuals (299 veterans with PTSD and 244 control subjects) [14], to the analysis of the effect of macromolecular crowding on the functionality of tumor suppressors ING containing a plant homeodomain (PHD) and their ability to interact with the histone H3 trimethylated at lysine 4 (H3K4me3) [15], and to the comparison of the effects of acute, chronic, and intermittent hypoxia on molecular pathways and cellular processes [16]. The breadth of the presented topics serves as a reflection of the wide diversity of the research areas dedicated to biomolecules. 
